# Evaluation of the short-term outcomes of robotic-assisted radical resection for perihilar cholangiocarcinoma: a propensity-scored matching analysis

**DOI:** 10.1093/gastro/goad018

**Published:** 2023-04-10

**Authors:** Xi-Tai Huang, Jin-Zhao Xie, Jian-Peng Cai, Wei Chen, Liu-Hua Chen, Li-Jian Liang, Xiao-Yu Yin

**Affiliations:** Department of Pancreato-Biliary Surgery, The First Affiliated Hospital, Sun Yat-sen University, Guangzhou, Guangdong, P. R. China; Department of Pancreato-Biliary Surgery, The First Affiliated Hospital, Sun Yat-sen University, Guangzhou, Guangdong, P. R. China; Department of Pancreato-Biliary Surgery, The First Affiliated Hospital, Sun Yat-sen University, Guangzhou, Guangdong, P. R. China; Department of Pancreato-Biliary Surgery, The First Affiliated Hospital, Sun Yat-sen University, Guangzhou, Guangdong, P. R. China; Department of Pancreato-Biliary Surgery, The First Affiliated Hospital, Sun Yat-sen University, Guangzhou, Guangdong, P. R. China; Department of Pancreato-Biliary Surgery, The First Affiliated Hospital, Sun Yat-sen University, Guangzhou, Guangdong, P. R. China; Department of Pancreato-Biliary Surgery, The First Affiliated Hospital, Sun Yat-sen University, Guangzhou, Guangdong, P. R. China

**Keywords:** robotic-assisted surgery, perihilar cholangiocarcinoma, biliary reconstruction

## Abstract

**Background:**

The application of robotic-assisted radical resection in perihilar cholangiocarcinoma (pCCA) remains poorly defined. This study aimed to evaluate the safety and efficacy of robotic-assisted radical resection for pCCA in our institute.

**Methods:**

Between July 2017 and July 2022, pCCA patients undergoing robotic-assisted and open radical resection at First Affiliated Hospital of Sun Yat-sen University (Guangzhou, China) were included. The short-term outcomes were compared by using propensity-scored matching (PSM) analysis.

**Results:**

Eighty-six pCCA patients were enrolled. After PSM at a ratio of 1:2, 10 and 20 patients were assigned to the robotic-assisted and open groups, respectively. There were no significant disparities in the clinicopathological features between the two groups. The robotic-assisted group had significantly longer operation time (median: 548 vs 353 min, *P *=* *0.004) and larger total number of lymph nodes examined (median: 11 vs 5, *P *=* *0.010) than the open group. The robotic-assisted group tended to have a lower intraoperative blood loss (median: 125 vs 350 mL, *P *=* *0.067), blood transfusion rates (30.0% vs 70.0%, *P *=* *0.056), and post-operative overall morbidities (30.0% vs 70.0%, *P *=* *0.056) than the open group, even though the differences were not statistically significant. There were no significant differences in the negative resection margin, post-operative major morbidities, or post-operative length-of-stay between the robotic-assisted and open groups (all *P *>* *0.05).

**Conclusions:**

Robotic-assisted radical resection of pCCA may get a larger total number of lymph nodes examined than open surgery. Provided robotic-assisted surgery may be a feasible and safe technique for selected pCCA patients.

## Introduction

Perihilar cholangiocarcinoma (pCCA) is a malignancy located in the hilar bile ducts, which accounts for ∼50% of all cholangiocarcinoma [1]. Radical resection, including extrahepatic bile duct resection, regional lymphadenectomy, and hepatectomy if necessary, remains the best hope to cure the disease. However, due to the locally advanced or metastatic disease at the time of exploration, the radical resection rate remains low [2, 3]. The prognosis of pCCA is poor, with a post-operative 5-year survival rate of ∼24%–30% [4–7].

Previous study has suggested that minimally invasive radical resection of pCCA might be comparable to open surgery [8]. However, the application of a minimally invasive technique in the radical resection for pCCA was hindered due to the difficulties in curative resection for pCCA, including the complex hilar anatomy, resection plane of hepatectomy, and tiny biliary reconstruction [9].

In recent years, a robotic-assisted surgical system has been gradually used in hepatobiliary surgery [10]. The robotic-assisted approach is technically more flexible and stable than the conventional laparoscopic approach due to its highly magnified naked-eye 3D vision and stable simulation manipulator, which is especially useful in performing complex biliary surgery [11]. However, the values of robotic-assisted radical resection for pCCA still remain controversial. One previous study reported that robotic-assisted surgery for pCCA significantly raised the occurrence rates of post-operative complications [12].

In this study, we used propensity-scored matching (PSM) analysis to evaluate the safety and feasibility of a robotic-assisted approach in radical resection for pCCA in comparison with those of an open approach in our single center.

## Patients and methods

### Patient selection

Patients with pCCA who underwent radical resection for pCCA via robotic-assisted and open approach at the First Affiliated Hospital of Sun Yat-sen University (Guangzhou, China) between July 2017 and July 2022 were included in this study.

**Figure 1.**
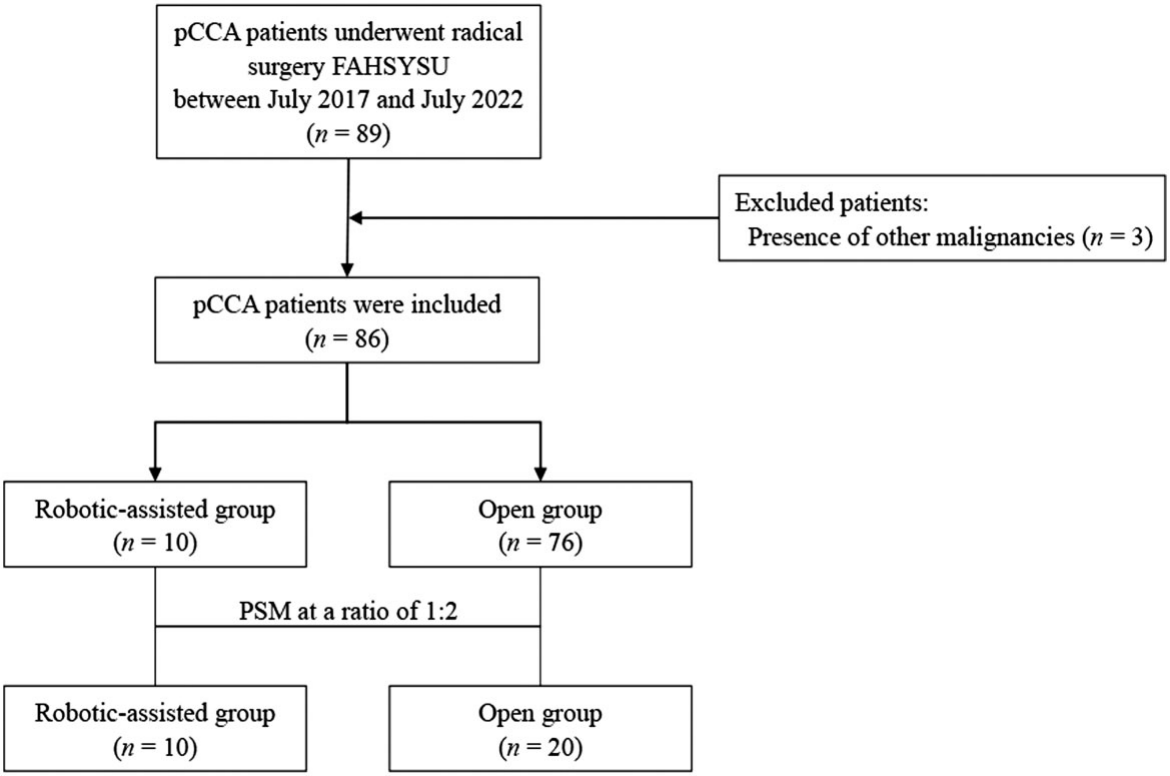
Flow chart of patient selection. PCCA, perihilar cholangiocarcinoma; FAHSYSU, First Affiliated Hospital of Sun Yat-sen University; PSM, propensity-scored matching.

The inclusion criteria included (i) preoperatively diagnosed as pCCA and confirmed by post-operative pathology; (ii) treated with robotic-assisted or open radical resection for pCCA; and (iii) absence of distant metastasis. The patients with co-existence of other malignancies were excluded. The flow chart is shown in Figure 1. This study was approved by the Ethics Committee of the First Affiliated Hospital of Sun Yat-sen University (Approval number: [2022]555).

### Surgical technique

**Figure 2.**
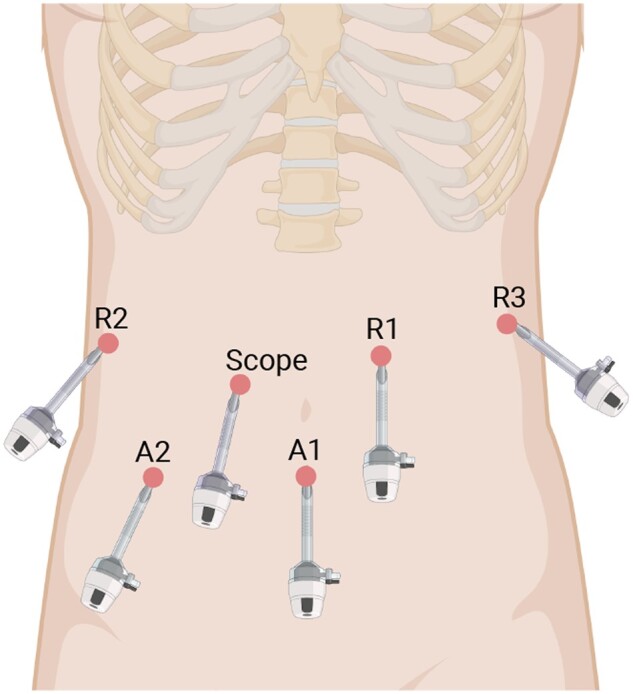
Trocar placement in robotic-assisted radical resection for perihilar cholangiocarcinoma. R1, 8-mm trocar for the first robotic arm; R2, 8-mm trocar for the second robotic arm; R3, 8-mm trocar for the third robotic arm; A1-A2, 12-mm trocars for assistant instruments.

All robotic-assisted procedures were performed using da Vinci Si Surgical System (Intuitive Surgical, Inc., Sunnyvale, USA) with four articulating robotic arms. Trocar placement of robotic-assisted radical resection for pCCA is shown in Figure 2. The surgical procedures were described as follows:

- Skeletonization of the hepatoduodenal ligament: The hepatoduodenal ligament was skeletonized to remove all soft tissues and lymph nodes, including Groups 8, 12, and 13 lymph nodes; the common hepatic artery, proper hepatic artery, common bile duct (CBD), and portal vein were adequately isolated.
- Transection of the distal CBD: The distal CBD was transected at the superior border of the pancreas and the cutting end was confirmed to be negative by the intraoperative frozen section.
- For Bismuth–Corlette type I, the extrahepatic bile duct with tumor and gallbladder was resected at the confluence of the right and left hepatic ducts, and the proximal cutting end was confirmed to be negative by the intraoperative frozen section. The left and right hepatic ducts were used for reconstruction of hepaticojejunostomy.
- For Bismuth–Corlette type III or IV, according to the predominant side of the cholangiocarcinoma, the ipsilateral hepatic artery and the portal vein were ligated and divided.
- Hemihepatectomy with caudate lobectomy: The hepatic parenchyma was gradually transected to adequately expose the hilar bile duct. The contralateral side of the hepatic duct was divided and the cutting end was confirmed to be negative by the intraoperative frozen section. The ipsilateral hepatic vein was transected to remove the hemi-liver with the caudate lobe, tumor, extrahepatic bile duct, and gallbladder. Complete hemostasis was achieved before reconstruction.
- Hepaticojejunostomy reconstruction: The modified loop-type hepaticojejunostomy was adopted for biliary reconstruction. End-to-side hepaticojejunostomy was performed by using continuous or interrupted suture according to the size of the hepatic duct. The hepaticojejunostomy proximal afferent jejunum was ligated, and side-to-side jejunostomy between hepaticojejunostomy afferent and efferent jejunum was performed by using continuous suture.
- Drainage placement and closure of the wound: Drainage tubes were routinely placed close to the hepaticojejunostomy and the cutting face of the liver, respectively. The tumor specimens were removed and the wound was closed.

### Data collection and perioperative management

Clinicopathological data of patients were retrospectively recorded. All patients were evaluated preoperatively by using at least two imaging investigations, including contrast-enhanced computed tomography, contrast-enhanced magnetic resonance imaging, and contrast-enhanced ultrasonography. The diagnosis and operative patterns were determined by the multidisciplinary treatment (MDT) team. Preoperative biliary drainage was performed if marked hyperbilirubinemia was presented. Patients were treated with robotic-assisted or open radical resection for pCCA.

The Bismuth–Corlette classification of the patient was determined by using the intraoperative finding and post-operative pathology. The outcomes measured included the operation time, intraoperative blood loss, blood transfusion, total number of lymph nodes examined (TNLE), resection margin, post-operative morbidity and mortality, and post-operative length-of-stay (LOS). The severity of the post-operative complication was evaluated by using Clavien–Dindo classification [13] and Clavien–Dindo III–IV were defined as major complications.

### PSM analysis

PSM, which has been widely used in the medical literature [14], was performed to minimize the bias between the robotic and open groups in the study. In this study, potential risk factors associated with the short-term and long-term outcomes after radical resection in pCCA were matched, including age, sex, body mass index (BMI), American Society of Anesthesiologists (ASA) classification, preoperative hemoglobin level, co-morbidities (hypertension and diabetes), preoperative biliary drainage, tumor size, tumor stage, and Bismuth–Corlette classifications. The propensity score for each patient was calculated through logistic regression modeling and then patients were matched at a ratio of 1:2 by using the method of “nearest.” The baseline features of patients were adjusted to balance after matching.

### Statistical analysis

The categorical variables are presented as frequencies with percentages and the continuous variables are presented as medians with interquartile range (IQR). Differences between categorical variables were compared by using the chi square test or Fisher’s exact test. Differences between continuous variables were compared by using the Mann–Whitney *U* test. PSM analysis was performed by using the “MatchIt” package [15]. Two-tailed *P *<* *0.05 was considered statistically significant. All statistical analyses were performed using SPSS version 24.0 software (IBM, Inc., Armonk, NY, USA) and R version 4.0.0 (http://www.Rproject.org).

## Results

### Clinicopathological features of patients undergoing radical resection for pCCA

A total of 86 patients who underwent radical resection for pCCA were enrolled, including 10 patients treated with robotic-assisted resection and 76 patients treated with open surgery. Of 10 patients treated with robotic-assisted surgery, 1 patient was converted to open surgery (conversion rate: 10.0%).

The clinicopathological features of patients were summarized and compared (Table 1). After PSM at a ratio of 1:2, 10 patients were assigned to the robotic-assisted group and 20 patients were assigned to the open group. There was no significant disparity between the robotic-assisted and open approach groups, including age, gender, BMI, ASA classification, co-morbidities (diabetes and hypertension), preoperative biliary drainage, Bismuth–Corlette classifications, tumor size, and T stage.

**Table 1.** Comparison of clinicopathological characteristics of patients treated with robotic-assisted and open radical resection for perihilar cholangiocarcinoma

Feature	Before PSM			After PSM		
	Robotic-assisted group (*n *=* *10)	Open group (*n *=* *76)	*P*-value^a^	Robotic-assisted group (*n *=* *10)	Open group (*n *=* *20)	*P*-value^a^
Age, years, median (IQR)	68 (59–71)	62 (52–70)	0.272^b^	68 (59–71)	66 (55–74)	1.000^b^
Sex, male, *n* (%)	7 (70.0%)	47 (61.8%)	0.738	7 (70.0%)	12 (60.0%)	0.702
BMI, kg/m^2^, median (IQR)	21.5 (20.7–22.6)	22.3 (20.4–24.2)	0.360^b^	21.5 (20.7–22.6)	22.2 (20.4–24.0)	0.373^b^
ASA classification ≥III, *n* (%)	7 (70.0%)	30 (39.5%)	0.092	7 (70.0%)	15 (75.0%)	1.000
Hemoglobin, g/L, median (IQR)	110 (103–132)	120 (108–129)	0.590^b^	110 (103–132)	116 (104–126)	1.000^b^
Hypertension, *n* (%)	2 (20.0%)	17 (22.4%)	1.000	2 (20.0%)	3 (15.0%)	1.000
Diabetes, *n* (%)	0 (0%)	6 (7.9%)	1.000	0 (0%)	0 (0%)	NA
Preoperative biliary drainage, *n* (%)	9 (90.0%)	55 (72.4%)	0.441	9 (90.0%)	15 (75.0%)	0.633
Bismuth–Corlette classification, *n* (%)			0.060^c^			1.000
I + II	5 (50.0%)	17 (22.4%)		5 (50.0%)	9 (45.0%)	
III + IV	5 (50.0%)	59 (77.6%)		5 (50.0%)	11 (55.0%)	
Tumor size, cm, median (IQR)	2.2 (1.5–3.2)	3.0 (2–3.5)	0.188^b^	2.2 (1.5–3.2)	2.0 (2.0–3.0)	0.914^b^
AJCC T stage, *n* (%)			1.000			1.000
T1 + T2	9 (90.0%)	62 (81.6%)		9 (90.0%)	18 (90.0%)	
T3 + T4	1 (10.0%)	14 (18.4%)		1 (10.0%)	2 (10.0%)	

a Fisher’s exact test.

b Mann-Whitney *U* test.

c Chi square test.

PSM, propensity score matching; IQR, interquartile range; BMI, body mass index; ASA, American Society of Anesthesiologists; AJCC, American Joint Committee on Cancer.

**Figure 3.**
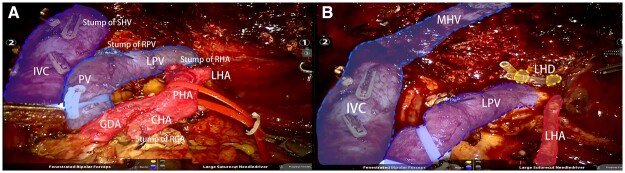
Intraoperative images of robotic-assisted radical resection for perihilar cholangiocarcinoma with Bismuth–Corlette type IIIA. (A) Images of skeletonization resection of the hepatoduodenal ligament. (B) Images of right hemihepatectomy with total caudate lobectomy. LHA, left hepatic artery; RHA, right hepatic artery; PHA, proper hepatic artery; CHA, common hepatic artery; RGA, right gastric artery; GDA, gastroduodenal artery; PV, portal vein; IVC, inferior vena cava; MHV, middle hepatic vein; RPV, right portal vein; LPV, left portal vein; LHD, left hepatic duct; SHV, short hepatic vein.

In the robotic-assisted group, seven patients were males and three patients were females, with a median age of 68 (59–71) years. The median tumor size was 2.2 (1.5–3.2) cm. Five patients were Bismuth type I or II and the remaining five patients were Bismuth type III or IV. All patients were treated with extrahepatic resection, regional lymphadenectomy, and hepatectomy if necessary (Figure 3).

In the open surgery group, 9 patients were Bismuth type I or II and the remaining 11 patients were Bismuth type III or IV.

### Comparison of short-term outcomes of patients treated with radical resection for pCCA

After PSM, short-term outcomes of patients treated with robotic-assisted and open radical resection for pCCA were compared (Table 2).

**Table 2.** Surgical details and post-operative outcomes of patients treated with robotic-assisted and open radical resection for perihilar cholangiocarcinoma

Feature	Robotic-assisted group (*n *=* *10)	Open group (*n *=* *20)	*P*-value^a^
Surgery type, *n* (%)			1.000
Hepatectomy with extrahepatic duct resection	5 (50.0%)	16 (53.3%)	
Extrahepatic duct resection alone	5 (50.0%)	14 (46.7%)	
Caudate lobectomy, *n* (%)	5 (50.0%)	12 (40.0%)	0.429
Operation time, min, median (IQR)	548 (434–711)	353 (284–523)	0.004^b^
Intraoperative blood loss, mL, median (IQR)	125 (50–425)	350 (100–600)	0.067^b^
Blood transfusion, *n* (%)	3 (30.0%)	14 (70.0%)	0.056
TNLE, median (IQR)	11 (6–13)	5 (3–9)	0.010^b^
Resection margin, R0, *n* (%)	9 (90.0%)	16 (80.0%)	0.640
Post-operative LOS, days, median (IQR)	12 (9–15)	15 (11–23)	0.155^b^
Overall morbidity, *n* (%)	3 (30.0%)	14 (70.0%)	0.056
Major morbidity^c^, *n* (%)	2 (20.0%)	10 (50.0%)	0.235
30-day mortality, *n* (%)	1 (10.0%)	0 (0%)	0.333

a Fisher’s exact test.

b Mann-Whitney *U* test.

c Clavien–Dindo classification grade III–V.

IQR, interquartile range; TNLE, total number of lymph nodes examined; LOS, length-of-stay.

The robotic-assisted group had significantly longer operation times (median: 548 vs 353 min, *P *=* *0.004) and higher TNLE (median: 11 vs 5, *P *=* *0.010) than the open group. Besides, the robotic-assisted group tended to have a lower intraoperative blood loss (median: 125 vs 350 mL, *P *=* *0.067), blood transfusion rates (30.0% vs 70.0%, *P *=* *0.056), and post-operative overall morbidities (30.0% vs 70.0%, *P *=* *0.056) than the open group, even though the differences were not statistically significant.

There was no significant difference in the negative resection margins (90.0% vs 80.0%, *P *=* *0.640), post-operative major morbidities (20.0% vs 50.0%, *P *=* *0.235), or post-operative LOS (median: 12 vs 15 days, *P *=* *0.155) between the robotic-assisted and open groups.

## Discussion

Radical resection for pCCA is a very challenging surgery, which is mostly performed via a conventional open approach. A minimally invasive technique has been widely used in the hepatobiliary surgery in recent years, including laparoscopic and robotic-assisted surgery. However, the application of minimally invasive surgery, especially a robotic-assisted approach, in the radical resection of pCCA remains in the preliminary exploratory stage. Since the first robotic-assisted radical resection for pCCA in our center in 2017, 14 cases of fully robotic-assisted surgeries for pCCA have been performed, but the post-operative pathology of 4 patients was not pCCA, so only 10 patients were finally assigned to the robotic-assisted group in this study.

By using PSM analysis for controlling some disparate risk factors, we found that although the robotic-assisted approach was associated with longer operation time, it could achieve higher TNLE than the conventional open approach. Besides, the robotic-assisted group tended to have a lower intraoperative blood loss, blood transfusion rates, and post-operative overall morbidities than the open group, even though the differences were not statistically significant. There were no significant differences in the negative resection margins, post-operative major morbidities, or post-operative LOS between the robotic-assisted and open groups. The current study was important because it indicated the potential safety and feasibility of robotic-assisted radical resection for pCCA.

Data on robotic-assisted surgery for pCCA have been limited to small case series and individual case reports until now [16]. Besides, these studies were all non-randomized–controlled studies with widely varying results. In 2016, by comparing 10 robotic-assisted with 32 open radical resections for pCCA, Xu *et al*. [12] found that the robotic-assisted surgery had significantly higher operation time and complication rates than the open surgery, whereas there were no significant differences in blood loss, post-operative LOS, or 90-day post-operative mortality between the two groups. Recently, a single-center study reported 48 robotic-assisted radical resections for pCCA, which was the largest number of cases reported to date but failed to compare with the open surgery [17]. In the robotic-assisted surgery group, the intraoperative blood loss was 150 mL, which was larger than that in this study; the total complication rate was 58.3%, which was higher than that in the present study. In addition, by including 101 robotic-assisted radical resections for pCCA, a meta-analysis showed that in the robotic-assisted group, the R0 resection rate was 71.0%, the average operation time was 660.8 min, the intraoperative blood loss was 188.5 mL, the post-operative LOS was 13.7 days, the post-operative complication rate was 61.3%, and the perioperative mortality was 2.0% [18]. In summary, the short-term outcomes of robotic-assisted radical resection for pCCA in this study were better than those of the previously reported studies.

However, data on the long-term outcomes of robotic-assisted radical resection of pCCA are still lacking. A previous study reported that the median tumor-free survival of pCCA patients treated with robot-assisted radical resection was 15.5 months (range, 6–60 months) [12]. However, most studies on robotic-assisted radical resection of pCCA only analysed the short-term outcomes [17, 19]. Therefore, more large randomized–controlled trials are still needed to evaluate its long-term efficacy, especially compared with traditional open surgery or laparoscopic surgery.

Although robotic-assisted radical resection of pCCA is very demanding, it is considered to have the following advantages compared with open and laparoscopic surgery. First, it facilitates thorough regional lymphadenectomy [20]. The magnification vision of the robotic surgical system can help find suspicious lymph nodes more easily, thereby resulting in skeletonization resection of the hepatoduodenal ligament (Figure 3A), which is consistent with the results of the present study. Second, it facilitates caudate lobectomy for most pCCA to improve the R0 resection rate, except for Bismuth–Corlette type I [21]. The magnified stereoscopic vision and increased wrist freedom allow easy dissection of the short hepatic veins from the caudate lobe to the bilateral sides of the inferior vena cava, which is useful for subsequent caudate lobectomy and reduces hemorrhage (Figure 3B). Third, it facilitates the reconstruction of small hepatic ducts. Multiple tiny hepatic duct openings on the liver-sparing side are often presented in radical resection of pCCA with Bismuth–Corlette type III and IV [22]. Robotic-assisted surgery facilitates bile duct reshaping and subsequent choledochojejunostomy or hepaticojejunostomy [23]. Specifically, no anastomotic bile leakage was found in the robot-assisted group in the present study. However, robotic-assisted surgery has some drawbacks, including high surgical costs, a lack of force feedback, and the need for a certain number of cases to overcome the learning curve.

This study has several limitations. First, this study was a single-center retrospective study with a relatively small sample size, which may lead to biased results. Second, due to the late development of the robotic-assisted radical resection for pCCA in our center, data on long-term efficacy were not available. The effectiveness of robotic-assisted radical resection for pCCA needs to be further explored by large prospective multicenter trials.

## Conclusions

The present study revealed that robotic-assisted radical resection of pCCA yielded larger TNLE than open surgery. Although robotic-assisted surgery had a longer operation time, it tended to have lower intraoperative blood loss, blood transfusion rates, and post-operative morbidities than open surgery. It represents one feasible and safe technique for selected pCCA patients. Its effectiveness in the short-term and long-term outcomes of pCCA needs to be further explored.

## Authors’ Contributions

Guarantor, conception, and design: X.Y.Y. Collection and assembly of data: X.T.H., J.Z.X., J.P.C., W.C., L.H.C., L.J.L. Data analysis and interpretation: X.T.H., J.Z.X., X.Y.Y. Manuscript writing and revision: X.T.H., J.Z.X., X.Y.Y. All authors read and approved the final manuscript.
